# Studies on the anticonvulsant potential of 6-gingerol, an active ingredient of ginger rhizome

**DOI:** 10.3389/fphar.2026.1740324

**Published:** 2026-04-23

**Authors:** Dorota Nieoczym, Wirginia Kukula-Koch, Małgorzata Szafarz, Jonathan Vuong, Anna Grenda, Dominik Tarabasz, Mateusz Pierog, Marta Marszalek-Grabska, Michal Szulc, Camila V. Esguerra, H. Steve White, Kinga Gawel

**Affiliations:** 1 Department of Functional Anatomy and Cytobiology, Institute of Biological Sciences, Faculty of Biology and Biotechnology, Maria Curie-Skłodowska University, Lublin, Poland; 2 Department of Pharmacognosy with Medicinal Plants Garden, Medical University of Lublin, Lublin, Poland; 3 Department of Pharmacokinetics and Physical Pharmacy, Jagiellonian University Medical College, Kraków, Poland; 4 Department of Pharmacy, School of Pharmacy, Center for Epilepsy Drug Discovery, University of Washington, Seattle, WA, United States; 5 Department of Pneumonology, Oncology and Allergology, Medical University of Lublin, Lublin, Poland; 6 Department of Neurotoxicology, Institute of Rural Health, Lublin, Poland; 7 Department of Pharmacology and Toxicology, Collegium Medicum, University of Zielona Gora, Zielona Gora, Poland; 8 Chemical Neuroscience Group, Norwegian Centre for Molecular Biosciences and Medicine, University of Oslo, Oslo, Norway; 9 Department of Pharmacology, Poznan University of Medical Sciences, Poznan, Poland

**Keywords:** 6-gingerol, anticonvulsant activity, ginger, mouse, seizures, zebrafish

## Abstract

Ginger (*Zingiber officinale*), due to its diverse therapeutic properties, continues to attract researchers’ attention. One of these properties is its anticonvulsant activity, which, as our previous studies have shown, may result from the presence of 6-gingerol, a compound that reduced pentylenetetrazole (PTZ)-induced hyperlocomotion in zebrafish larvae. In the present study, we further investigated the anticonvulsant effects of 6-gingerol using additional seizure and epilepsy models in both zebrafish larvae and mice. First, we observed that 6-gingerol reduced the overexpression of the neuronal PAS domain protein (*npas4*) gene in PTZ-exposed zebrafish larvae, but it did not affect the expression of brain-derived neurotrophic factor (*bdnf*). The anticonvulsant effect of 6-gingerol was evident in the ethyl 2-ketopent-4-enoate seizure test in zebrafish larvae but was not observed in the pilocarpine model or in *scn1lab* (Dravet syndrome model) and *cacna1aa* (absence seizure model) zebrafish morphants. In mice, 6-gingerol penetrated the blood-brain barrier and inhibited psychomotor seizures induced by 6 Hz electrical stimulation at an intensity of 32 mA, but it was ineffective at 44 mA. We did not observe any anticonvulsant effects of 6-gingerol in the maximal electroshock test or timed intravenous PTZ infusion seizure test in mice. In summary, our findings provide further evidence for the anticonvulsant effects of 6-gingerol; however, additional investigations are required to clarify its mechanisms of action.

## Introduction

Epilepsy is a chronic disease that results from abnormal and synchronized discharges of neurons in the central nervous system. It affects about 70 million people worldwide, which makes it one of the most prevalent neurological diseases (Thijs et al., 2019). Epileptic syndromes are often characterized by seizures that might have diverse symptomatology dependent on the brain region affected by abnormal electrical activity of neurons–from brief disturbances of consciousness, state of confusion and disorientation, through some vegetative (e.g., changes in blood pressure, sweating, changes in heart rate) and sensory (e.g., itching, olfactory or visual hallucinations) disturbances to motor seizures (myoclonic, clonic, tonic or atonic).

Diagnosis of epilepsy is challenging due to the varied symptomatology and etiology of the disease. In order to achieve the best seizure control, it is essential to select adequate and effective treatment. Despite significant advancement in antiseizure medication (ASM) development, approximately 30% of patients do not respond to pharmacotherapy, which is referred as drug-resistant or refractory epilepsy (Belete, 2023; Löscher et al., 2013). ASMs available on the pharmaceutical market act only symptomatically and do not prevent epilepsy development in high-risk population, nor reverse changes induced in the brain by epileptogenesis process. Therefore, enhancing the efficacy of epilepsy treatment necessitates the development of pharmacological agents that specifically target the molecular and cellular mechanisms underlying ictogenesis and epileptogenesis. Furthermore, the creation of safer medications remains imperative, as debilitating side effects during pharmacotherapy prompt patient discontinuation, even if it result in a significant reduction in occurrence of seizures (Löscher et al., 2020; Łukasiuk and Lasoń, 2023).

Natural plant-derived compounds continue to be investigated as a potential source of new ASMs; e.g., cannabidiol (Martimbianco et al., 2025; Moreira et al., 2024; Borowicz-Reutt et al., 2024) and huperzine A (Damar et al., 2016). Additionally, extracts and compounds from ginger rhizomes have attracted the interest of researchers. Numerous studies have revealed that compounds isolated from ginger, i.e., 6-gingerol, have the ability to cross the blood-brain barrier (Simon et al., 2020; Lim et al., 2025; Kuswandani et al., 2025) and thereby might affect the central nervous system activity. Our previous studies revealed anticonvulsant properties of 6-gingerol isolated from *Zingiber officinale* rhizome extract in the pentylenetetrazole (PTZ) seizure test in the zebrafish larvae (Gawel et al., 2021). PTZ-induced seizures serve as a common model in early preclinical studies using rodents and zebrafish to identify compounds with anticonvulsant activity (Rubio et al., 2024). 6-gingerol decreased PTZ-induced hyperlocomotion, which considered a marker of convulsant activity in the zebrafish larvae, as well as restricted the occurrence of local field potential (LFP) events detected in the *optic tectum* of larvae. Anticonvulsant effect of 6-gingerol determined based on behavioral and electroencephalographical (EEG) analysis was also associated with changes in γ-aminobutyric acid (GABA) and glutamate levels; i.e., 6-gingerol normalised PTZ-induced changes in the glutamate/GABA rate (Gawel et al., 2021).

The aim of the present study was to further evaluate the anticonvulsant properties of 6-gingerol and thereby provide a more accurate assessment of its therapeutic properties and potential utility as an epilepsy therapy. First, we verified our previous results from the PTZ seizure test in zebrafish larvae at the molecular level using RT-PCR analysis to assess changes in mRNA expression of two markers of neuronal activity, i.e., brain-derived *bdnf* and neuronal Per-Arnt-Sim (PAS) domain protein (*npas4*) genes. We continued our studies using seizure models in zebrafish larvae, i.e., the ethyl 2-ketopent-4-enoate (EKP)- and pilocarpine (PILO)-induced seizure tests as well as *scn1lab cacna1aa* and three experimental models in mice, i.e., intravenous (i.v.) PTZ seizure threshold, maximal electroshock and 6-Hz induced psychomotor seizure tests. Moreover, the effect of 6-gingerol on motor coordination (assessed in the chimney test) and neuromuscular strength (assessed in the grip strength test) was evaluated to estimate possible side effects that might significantly limit the therapeutic use of this compound.

## Materials and methods

### Drugs and reagents

PTZ, pilocarpine hydrochloride (PILO), and sodium valproate (VPA) were purchased from Sigma Aldrich (St. Louis, MO, United States) and dissolved in miliQ water (miliQW) or saline (0.9% NaCl), on the day of their usage. Ethyl 2-ketopent-4-enoate (EKP), mixed with miliQW, was synthesized by dr. Tomasz Wróbel (Medical University of Lublin, Poland) as described in detail previously (Nieoczym et al., 2024). The isolation of 6-gingerol is described in the Supplementary Material.

### Zebrafish husbandry and ethical statement

For all zebrafish experiments (Medical University of Lublin, Poland and Centre for Molecular Medicine Norway, University of Oslo, Norway), larval zebrafish of AB strain up to 5 days post-fertilization (dpf) were used. Embryos and subsequently larvae were reared in standard aquaculture conditions (at incubator: 28.5 °C ± 0.5 °C, 14/10 h light to dark cycle) in artificial zebrafish water (E3 medium) that consisted of 1.5 mM HEPES, pH 6.5, 17.4 mM NaCl, 0.21 mM KCl, 0.12 mM MgSO_4_, 0.18 mM Ca(NO_3_)_2_. Fish water was replaced every day until experiments started (day 3 or 4).

In accordance with current European legislation, ethical permission to conduct assays in Poland was not required since larvae up to 5 dpf were used. For experiments conducted in Norway (morpholino injections and LFP recordings), approval of the Norwegian Food Safety Authority (FOTS-ID 30816) was obtained. In either case, husbandry and the experimental procedures were performed in compliance with the National Institute of Health Guidelines for the Care and Use of Laboratory Animals and the European Union Directive of 22 September 2010 (2010/63/EU) on protecting animals used for scientific purposes and ARRIVE guidelines. All efforts were taken to minimize larval stress and suffering. Tricaine solution (15 μM) was used for larvae euthanasia if applicable. Wherever possible, the analysis was conducted by observer blind to treatment groups.

### EKP- and PILO-induced seizure assays in larval zebrafish

The experiments were conducted as previously described (Gawel et al., 2024; Nieoczym et al., 2024). Briefly, 4-day old larvae were incubated in varying concentrations of 6-gingerol (12.5, 25, 31.25 or 37.5 μM) for 22 h in 48-well plates in incubator (28.5 °C ± 0.5 °C). Each well contained 1 larva. Behavioural tracking was performed at 28.5 °C under controlled illumination. 6-gingerol was dissolved in pure DMSO to obtain stock and diluted in embryo medium before usage. The final concentration of DMSO did not exceed 0.2% in final solution. Medium supplemented with equivalent concentration of DMSO served as vehicle.

The concentrations used were determined in our earlier study (Gawel et al., 2021). As a follow up, miliQW or chemoconvulsant (EKP at final concentration of 200 μM or PILO at final concentration of 50 mM) was added (Gawel et al., 2024; Nieoczym et al., 2024). After 2- (for PILO) or 5-min (for EKP) delay tracking in Noldus (Wagenigen, Netherlands) tracker device was started. The whole analysis took 30 (for EKP) or 28 min (for PILO). EthoVision XT software (Noldus, Wageningen, Netherlands) was used for data collection and analysis. Distance traveled by larvae in mm was taken for analysis. All experiments were replicated three times and the data were pooled.

### Antisense morpholino oligomers injections

To achieve partial knockdown of desired genes, antisense morpholino oligomers (MOs) were purchased from GeneTools LLC (Philomath, Oregon, United States). They were injected into one- or two-cell stage zebrafish embryos. Zebrafish standard control MO (sequence 5′-CCT​CTT​ACC​TCA​GTT​ACA​ATT​TAT​A) was used as a control (9 ng, volume 1.5 nL per embryo). The sequence of *scn1lab* (zebrafish model of Dravet syndrome) MO was chosen from Zhang et al. (2015) (translation blocking MO: 5′-CTG​AGC​AGC​CAT​ATT​GAC​ATC​CTG​C-3′; 9 ng, volume 1 nL per embryo). The sequence of *cacna1aa* (zebrafish model of absence seizures) MO was chosen based on our previous study (Gawel et al., 2020) (targets for ATG codons of *cacna1aa-201* and *cacna1aa-202* and *cacna1aa-203* transcripts: 5′-TGT​ACT​CAA​TGG​AGT​GAG​AAT​CAT-3′ and 5′-TCA​TCT​CCG​AAC​CGA​GCC​ATT​CTA​T-3′, respectively). *cacna1aa* MOs were injected in the amount of 2.5 ng + 2.5 ng, total volume of 1.5 nL per embryo. The control group was the same in both experiments, i.e., with *cacna1aa* and *scn1lab* morphants.

### Assessment of LFP recordings in *scn1lab*- and *cacna1aa*-morphants

In order to assess the effect of 6-gingerol (concentration 37.5 μM) on epileptiform-like discharges in *scn1lab*- and *cacna1aa*-morphants, LFP recordings were conducted as described earlier (Gawel et al., 2020; Gawel et al., 2021; Gawel et al., 2024). Epileptiform-like events were defined as discharges exceeding three times the baseline noise level. Briefly, 3 dpf larvae were incubated in 6-gingerol for 22 h, and subsequently mounted in a glass slide in 2% low-melting point agarose (Sigma-Aldrich, United States). Following this, a hollow glass electrode (1–5 MΩ) was inserted into the brain of 4 dpf larva (exactly into the *optic tectum*, part of the midbrain) (MultiClamp 700B amplifier, Digidata 1550 digitizer, Axon instruments, United States). The artificial cerebrospinal fluid consisted of 124 mM NaCl, 2 mM KCl, 2 mM MgSO_4_, 2 mM CaCl_2_, 1.25 mM KH_2_PO_4_, 26 mM NaHCO3, and 10 mM glucose. Each recording lasted 20 min. Clampfit 10.20 software was used for data processing (Molecular Devices Corporation).

### Real-time PCR

RNA isolation was carried out using PureLink™ RNA Mini Kit (Thermo Fisher Scientific, Waltham, Massachusetts, United States) according to the manufacturer’s instruction. The reverse transcription reaction was done with High-Capacity cDNA Reverse Transcription Kit (Thermo Fisher Scientific, Waltham, Massachusetts, United States) according to the manufacturer’s instruction. A real-time PCR reaction was performed utilizing GoTaq® Master Mix (Promega, Madison, Wisconsin, United States). Ribosomal RNA (18S rRNA) and beta-2-microglobulin (b2m) were used as an internal control (housekeeping genes). Relative gene expression was calculated using the 2^−ΔΔCt^ method. The sequences of the primers used in real-time PCR are given in Table 1.

**TABLE 1:** The sequence of the respective primers used in the study.

Gene	Primers	Source
*npas4*	F: AGCCAAGTCTGCCCTTCTTCT R: TGCTGTGCTAAAAGCGAGATCT	Klarić et al. (2014)
*bdnf*	F: CGAGGAATAGACAAGCGGCA R: ATCCGTATAAACCGCCAGCC	Blanco et al. (2020)
*18s*	F: TCGCTAGTTGGCATCGTTTATG R: CGGAGGTTCGAAGACGATCA	McCurley and Callard (2008)
*b2m*	F: GCCTTCACCCCAGAGAAAGG R: GCGGTTGGGATTTACATGTTG	McCurley and Callard (2008)

### Mice

The maximal electroshock seizure (MES), 6 Hz psychomotor seizure and rotarod tests were conducted at the University of Washington (Seattle, United States) and employed male CF-1 mice (25–40 g; 5–6 weeks old; Charles River Laboratories). Mice were housed five mice/cage with corncob bedding in a temperature-controlled specific pathogen-free vivarium on a 14:10 light/dark cycle (on: 06:00 a.m., off: 8:00 p.m.). Animals were given free access to irradiated chow (Picolab 5053) and filtered water, except during periods of behavioural manipulation. Mice were allowed to acclimate to the housing facility for at least 5 days and to the testing room for at least 1 h prior to testing. All studies were conducted during the animals’ light phase. Animals were euthanized by CO_2_ asphyxiation. This study was not designed to assess the impact of sex as a biological variable; thus, only male mice were used. All animal use was approved by the University of Washington Institutional Animal Care and Use Committee, conformed to the Animal Research: Reporting of *In Vivo* Experiments (ARRIVE) guidelines and was conducted in accordance with the United States Public Health Service’s Policy on Humane Care and Use of Laboratory Animals.

Pharmacokinetic studies, intravenous (i.v.) PTZ seizure, grip-strength and chimney tests were carried out at Maria Curie-Skłodowska University in Lublin (Poland) using adult male Crl:CD1(ICR) mice (weight 25–35 g) purchased from Charles River (Sulzfeld, Germany). Animals were housed at temperature 21 °C–24 °C, relative humidity 45%–65%, 12 h light/12 h dark cycle (light on at 6:00 a.m.). The animals were provided with constant access to food pellets and fresh water. Mice were employed in experiments after at least 7-day quarantine and acclimatization to the housing rooms. All behavioural tests were conducted between 8:00 a.m. and 4:00 p.m. during the light phase of the light/dark cycle. Both housing and experimental procedures were conducted according to the guidance of the European Union Directive of 22 September 2010 (2010/63/EU), ARRIVE guidelines and Polish laws on animal experimentation. All experimental procedures were accepted by Local Ethical Committee in Lublin (licence no. 76/2024).

### Pharmacokinetic studies

6-gingerol was suspended in 1% Tween solution in saline (0.9% NaCl) and given intraperitoneally (i.p.) at a dose of 10 mg/kg (n = 5 per time point). Blood samples were collected at six time points, i.e. 15, 30, 45, 60, 120 and 360 min, following compound administration, and allowed to clot at room temperature for 20 min. Brains were removed from skulls and washed with 0.9% NaCl. The blood samples were centrifuged for 10 min at a speed of 8,000 rpm. The obtained serum and brains were stored at −80 °C until analysis. The determination of the 6-gingerol content in the biological material is described in the Supplementary Material.

### Pharmacokinetic data analysis

Pharmacokinetic parameters were calculated using the Monolix 2023R1 (free academic license), Lixoft SAS, a Simulations Plus company software employing a non-compartmental approach. The maximum concentration (C_max_) and the time to reach maximum concentration (t_max_) were obtained directly from the concentration *versus* time profile. The area under the mean serum concentration *versus* time curve extrapolated to infinity (AUC_0-inf_) was estimated using the log/linear trapezoidal rule and the extrapolated area calculated as C_last_/λ_z_ where C_last_ is the last measured concentration at the last sampling time (t_last_). Similarly, AUMC was estimated by calculation of total area under the first-moment curve by combining trapezoid calculation of AUMC_0-t_ and extrapolated area. The terminal slope (λ_z_) was calculated by log-linear regression of the drug concentration data in the terminal phase and the terminal half-life (t_1/2λz_) was calculated as 0.693/λ_z_. The volume of distribution based on the terminal phase (V_z_/F) was calculated as: Dose/(λ_z_ ⋅AUC_0-inf_) and clearance (CL/F) was obtained from the equation: Dose/AUC_0-inf_, where F is fraction of dose absorbed. The mean residence time (MRT) was calculated as: AUMC_0-inf_/AUC_0-inf_.

### Mouse seizure tests

Animals were adapted to housing conditions for at least 1 week before being used in the experiments. The experiments were conducted after 30- or 60-min acclimatisation to the laboratory rooms. Moreover, control and drug experiments were conducted on the same day to avoid day-to-day changes in the convulsive susceptibility.

### The maximal electroshock test

The MES test serves as a validated model for generalized tonic-clonic seizures in humans and represents a critical preclinical screening tool for assessing anticonvulsant drug candidates (Castel-Branco et al., 2009; White et al., 1995; Löscher et al., 1991). Fifteen minutes after i.p. administration of 6-gingerol (10 and 60 mg/kg), mice received transcorneal electrical stimulation at 50 mA (0.2 s duration; 60 Hz frequency) using an apparatus similar to that originally described by Woodbury and Davenport (Woodbury and Davenport, 1952). Immediately prior to the electroshock stimulation, a drop of anaesthetic (0.5% tetracaine) was applied to the eyes for local anaesthesia. Electrode tips were wetted with 0.9% saline immediately before stimulation to ensure optimal electrical conductivity and corneal contact. Mice were manually restrained during stimulus delivery, then transferred to observation cages for seizure monitoring. The MES-induced seizures were characterized by tonic hindlimb extension greater than 90° from the body plane. Protection was defined as the absence of tonic hindlimb extension. The number of animals in each experimental group was within the range of 6–11.

### The 6 Hz psychomotor seizure test

The 6 Hz-induced seizure test in mice is an experimental tool used to evaluate anticonvulsant substances for potential efficacy against partial seizures in humans (Barton et al., 2001). During the test, mice were manually restrained by the experimenter, and low-frequency electrical stimulation (6 Hz, 0.2 ms rectangular pulse width, 3-s duration) at 32 mA or 44 mA intensity was delivered through corneal electrodes connected to a constant-current electroshock stimulator (Grass S48 stimulator). Following stimulation, mice were placed in a plexiglass observation chamber to monitor behavioral responses. Psychomotor seizure symptoms include initial immobilization, vibrissae twitching, jaw and forelimb clonus, head nodding, rearing, and Straub tail. Animals not exhibiting convulsive behavior or displaying symptoms lasting fewer than 10 s are classified as “protected”, indicating potential drug efficacy. Experimental groups consisted of 6–11 mice.

### The i.v. PTZ test

PTZ, a GABA_A_ receptor antagonist, induces convulsions in experimental animals, providing a model for tonic-clonic seizures (Mandhane et al., 2007). Although PTZ-induced seizures can be observed following both subcutaneous and i.p. administration, the i.v. PTZ seizure threshold test is particularly valuable in preclinical studies of compounds with potential anticonvulsant properties. This method allows for the evaluation of the compound’s effect on the threshold for three types of seizures: myoclonic, generalized clonic, and tonic seizures (Mandhane et al., 2007). In our study, the i.v. PTZ seizure threshold test in mice was used to assess the impact of 6-gingerol on seizure thresholds.

For the PTZ test, each mouse was placed in a Plexiglas restrainer to facilitate lateral tail vein injection using a needle (27G, ¾ in., Sterican, B. Braun Melsungen AG, Melsungen, Germany) connected to a plastic syringe *via* a polyethylene tube (PE20RW, Plastics One Inc., Roanoke, VA, United States). The syringe contained a 1% solution of PTZ in saline (0.9% NaCl) was positioned in a syringe pump (Harvard Apparatus, Holliston, MA, United States). Proper needle insertion into the vein was confirmed by the presence of blood in the tube. To prevent needle dislocation during seizures, a piece of adhesive tape was applied. After injection, the mouse was removed from the restrainer and placed in a transparent box where it could move freely. The PTZ solution was administered into the vein at a constant rate of 0.2 mL/min. The cut off-time for infusion of PTZ solution was 180 s. The time from the start of infusion to the appearance of three types of seizures–myoclonic twitches, generalized clonic seizures with loss of balance, and tonic hind limb extension, was measured. The seizure thresholds, representing the amount of PTZ required to induce each type of seizure, were calculated using the formula: PTZ (mg/kg) = [infusion duration (s) × infusion rate (mL/s) × PTZ concentration (mg/mL)]/ weight (kg). Experimental groups consisted of 12–18 mice.

### The rotarod test

The rotarod test was conducted in mice subjected to the MES or 6 Hz tests to asses impairment in motor coordination. Animals were placed individually on a horizontal rod rotated about its long axis (6 rpm), for 1 min over 3 consecutive trials. Mice that did not show motor coordination disturbances were able to remain on the rod, while those with impaired coordination fell off in less than 60 s (Dunham and Miya, 1957).

### The chimney test

The chimney test is a simple method used for evaluation of motor coordination in rodents (Boissier et al., 2008). In the test, mice were allowed to enter a transparent Plexiglas tunnel (chimney) with an inner diameter of 3 cm and a length of 30 cm. Once a mouse reached its distal end, the chimney was set upright. Animals that failed to climb out of the chimney backwards within a maximum of 60 s were considered to have impaired motor coordination. Since the chimney test is non-invasive, it was performed immediately before the i.v. PTZ test. The number of mice in each experimental group was 16.

### The grip strength test

The grip strength test is used to evaluate neuromuscular functions in rodents based on the grip strength of the mouse’s forelegs (Meyer et al., 1979). The apparatus used for measuring the grip strength consists of a force transducer connected to a metal grid. The mouse, held by its tail, is brought close to the grid so that it can grasp it with its forelegs, and then is gently pulled back. The apparatus measures the maximum grip strength of the animal (in mN). The measurement for each animal is repeated three times and the results are normalized to body weight (g). The grip strength test was carried out immediately before the i.v. PTZ test, with the chimney test.

Results for each experimental group are presented as mean muscle strength + standard error of the mean (SEM). The number of mice in each experimental group was 16.

### Statistical analysis

The size of the experimental groups was determined at the planning phase, taking into account the power of statistical tests.

The results from all experiments were analyzed for outliers assessed only for normally distributed datasets.

Locomotor activity data obtained from zebrafish seizure assays were not normally distributed and were therefore analyzed using the nonparametric Kruskal–Wallis test followed by Dunn’s multiple comparisons test. Statistical inference was based primarily on total distance traveled, while time-bin data are presented descriptively to illustrate behavioral dynamics. Data are expressed as median with 95% confidence intervals.

Real-time PCR data were analyzed using one-way ANOVA followed by Tukey’s *post hoc* test. Assumptions of normality was verified prior to analysis. Results are presented as mean + SEM. Number of LFP events in *cacna1aa-*morphants and *scn1lab*-morphants were compared with Kruskal–Wallis test (due to the lack of normal distribution). Data from these two genetic models of epilepsy are presented as median with 95% confidence intervals.

Seizure incidence data from the MES and 6 HZ tests, as well as results from the chimney test were analyzed using Fisher’s exact test. Seizure threshold data from the i.v. PTZ test were analyzed using one-way ANOVA followed by Dunnett’s *post hoc* test for normally distributed variables, or Kruskal–Wallis test followed by Dunn’s *post hoc* test when normality assumptions were not met. Although overall group effects were evaluated, interpretation of treatment efficacy was based on *post hoc* comparisons *versus* control. Data from the i.v. PTZ test are presented as mean dose of PTZ + SEM or median with 95% confidence intervals.

Statistical analyses were hypothesis-driven for each experimental model; therefore, no global correction across models was applied. The level of statistical significance was set at p < 0.05. All tests were two-tailed.

GraphPad Prism 10 version (San Diego, CA, United States) was used for analysis and figure generation.

## Results

### The results of 6-gingerol isolation from the extract

**FIGURE 1:**
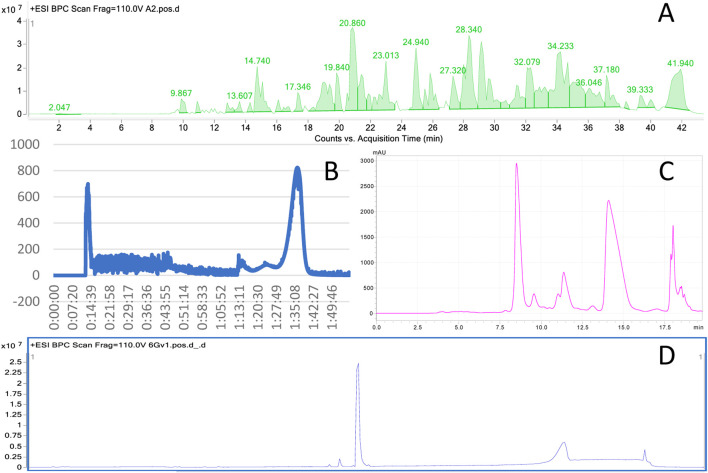
The total ion chromatogram from ginger rhizomes used for further fractionation with 6-gingerol at 20.86 min **(A)** CPC chromatogram obtained from the fractionation of ginger extract, recorded at 290 nm **(B)** A sample HPLC-DAD chromatogram recorded at 282 nm coming from the purification of 6-gingerol from the first peak in the CPC fractionation in a preparative HPLC chromatograph **(C)** The peak of 6-gingerol at 20.8 min in the total ion chromatogram recorded in positive ion mode as a result of preparative HPLC purification of a CPC fraction **(D)**.

6-Gingerol was identified in the prepared extract using the HPLC-MS technique by comparison with a solution of standard. Mass spectrometric analysis revealed that 6-gingerol eluted at 20.8 min under the described method and was among the most prominent components of ginger extract (Figure 1A).

The introduction of gradient separation into the CPC methodology provided satisfactory results for the separation of 6-gingerol from other constituents of this rich matrix of ginger extract. 6-Gingerol was washed out in the first peak after 12 min of separation–at the very beginning of the CPC chromatogram. This makes the methodology a beneficial one due to its short analysis time and reduced solvents consumption, as further injections aimed at 6-gingerol isolation were shortened and limited to the collection of the first eluted peak (Figure 1B). The fraction enriched in 6-gingerol obtained from CPC was further purified using semi-preparative HPLC. The collected fraction, as visualized by the analysis on a preparative HPLC chromatograph, contained four major components. A sample preparative HPLC chromatogram is presented in Figure 1C. The analysis was monitored at a wavelength of 282 nm under isocratic conditions (acetonitrile/water 65:35, v/v) with a flow rate of 12 mL/min. Further steps included separation of 6-gingerol from the collected mixture. The representative chromatogram (Figure 1D) showed a well-resolved peak corresponding to 6-gingerol, collected between 8 and 9 min. No significant co-eluting peaks were observed, indicating effective separation of 6-gingerol from other components of the fraction.

The identity and purity of the purified fraction were confirmed by HPLC-MS analysis (Figure 1D). The MS spectrum in ESI + mode displayed a single dominant peak at 20.8 min, with minimal background noise. The mass spectrum corresponding to this peak revealed [M–H_2_O + H]^+^ ion at m/z 277.1798, resulting from the loss of a water molecule from 6-gingerol (C_17_H_26_O_4_; calculated [M + H]^+^ 295.1904). This observation was further confirmed by analysis of an authentic 6-gingerol standard, which showed an identical fragmentation pattern. Based on peak area integration, the purity of the isolated 6-gingerol was estimated to exceed 98%.

These results demonstrate that the applied semi-preparative HPLC method enables efficient separation of 6-gingerol from other extract components and the reproducibility of retention times in both HPLC and LC-MS indicates the stability and reliability of the protocol.

In conclusion, the combination of semi-preparative HPLC and LC-MS provides a reliable and effective approach for obtaining high-purity 6-gingerol from natural sources. The applied chromatographic conditions (isocratic elution with acetonitrile/water 65:35, v/v, flow rate 12 mL/min, detection at 282 nm) proved to be optimal, allowing their further application for the isolation of 6-gingerol analogues.

### Effect of 6-gingerol on the seizure activity in the EKP-induced seizure model in larval zebrafish

Changes in the EKP-induced locomotor activity of 6-gingerol treated zebrafish larvae were assessed as total distance traveled within 30 min and analyzed using Kruskal–Wallis test with Dunn’s *post hoc* test (KW = 71.91, p < 0.0001). EKP at concentration of 200 µM significantly increased total distance traveled by zebrafish larvae in comparison to the control (vehicle + miliQW) group (p < 0.001). The most pronounced difference in locomotor activity between the control group and the EKP-incubated group was observed mainly during the first half of the experiment, while in the second half, the locomotor activity of the two groups did not differ as much. Zebrafish larvae incubated for 22 h with 6-gingerol at concentrations ranging from 12.5 to 31.25 µM did not show any limitation of EKP-induced locomotor activity (p > 0.05). Statistically significant changes in larvae locomotor activity were noted in group incubated with 6-gingerol at the highest tested concentration, i.e., 37.5 µM (p < 0.001).

**FIGURE 2:**
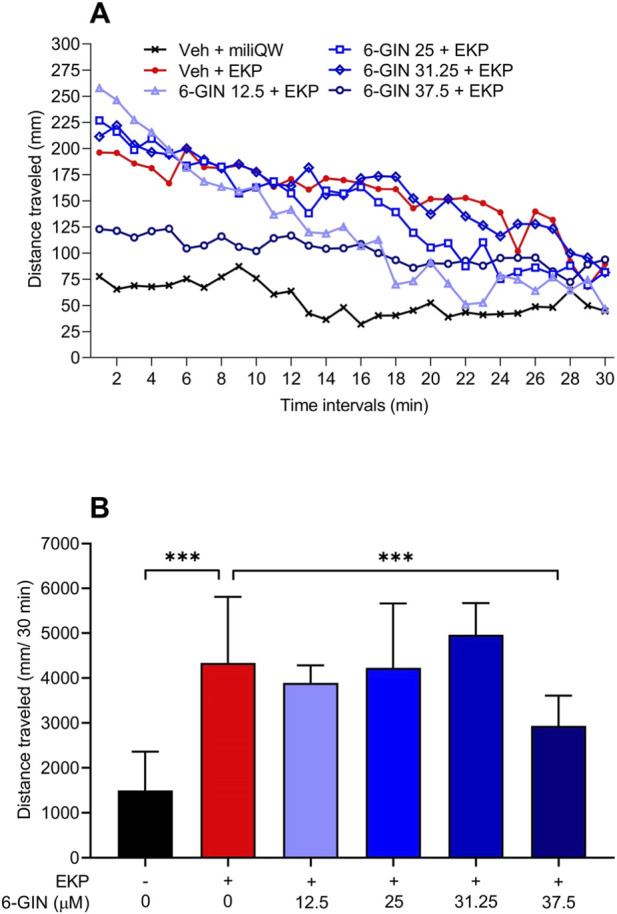
The effect of 6-gingerol (concentrations 12.5–37.5 μM) on hyperlocomotion in the EKP-induced (200 μM) seizure model in larval zebrafish. After 22 h long incubation in different 6-gingerol concentrations, larvae were exposed to acute dose of EKP, and after 5 min delay analysis started. Results of the experiments are shown as distance traveled in 2 min-long time bins **(A)** Time-bin analyses are presented descriptively; statistical inference marked was based on total distance traveled within 30 min of the assay **(B)**. Data on total distance traveled within 30 min **(B)** were analyzed using Kruskal–Wallis test with Dunn’s multiple comparison test: KW = 71.91, p < 0.0001. Data are shown as median with 95% confidence limits (n = 39–48 per group) ***p < 0.001. 6-GIN, 6-gingerol; EKP, ethyl 2-ketopent-4-enoate; miliQW, miliQ water; Veh, vehicle.

Effect of 6-gingerol on EKP-induced hyperlocomotion is illustrated in Figure 2.

### Effect of 6-gingerol on the seizure activity in the PILO-induced seizure test in larval zebrafish

**FIGURE 3:**
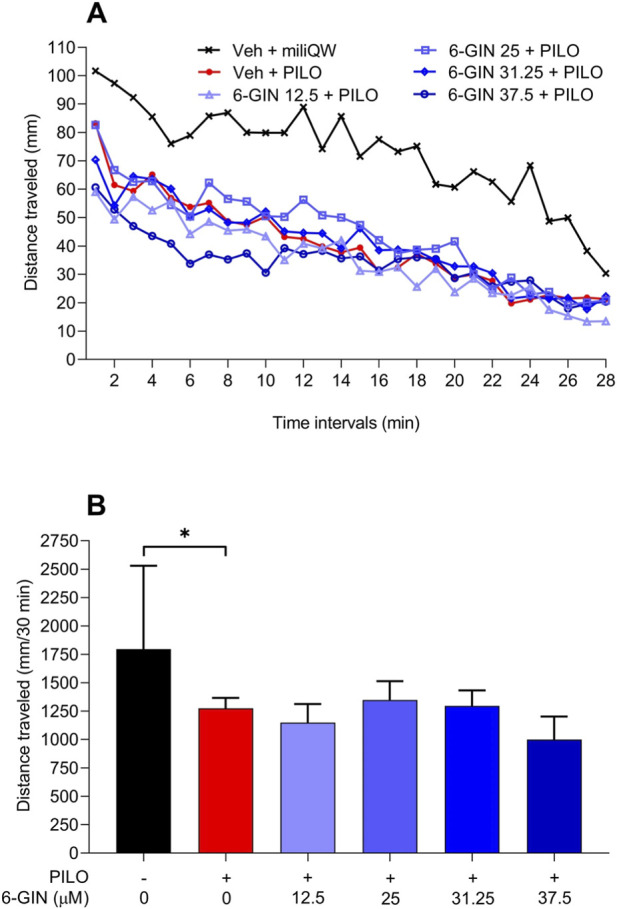
The effect of 6-gingerol on hypolocomotion in the PILO-induced seizure model in larval zebrafish. After 22 h long incubation in different 6-gingerol concentrations (12.5–37.5 μM) larvae were exposed to PILO (50 mM) and after 2 min delay analysis of locomotor activity started. Results of the experiments are shown as distance traveled in 2 min-long time bins **(A)**. Time-bin analyses are presented descriptively; statistical inference marked was based on total distance traveled within 28 min **(B)**. Data were analyzed using Kruskal–Wallis test with Dunn’s multiple comparison test: KW = 25.34, p < 0.001. Data are shown as median + 95% confidence limits (n = 32–48 per group). *p < 0.05. 6-GIN, 6-gingerol; miliQW, miliQ water; PILO, pilocarpine hydrochloride; Veh, vehicle.

Statistical analysis of the results revealed that PILO at concentration of 50 mM significantly limited locomotor activity in the zebrafish larvae (Figure 3). According to Gawel et al. (2024), PILO-induced hypolocomotion in zebrafish larvae is behavioral manifestation of seizures and it is correlated with abnormal electrical activity of the brain (Gawel et al., 2024). 6-gingerol pretreatment at concentrations ranging from 12.5 to 37.5 μM did not affect significantly locomotor activity in larval zebrafish treated with PILO (p > 0.05).

### Effect of 6-gingerol on *npas4* and *bdnf* mRNA expression in zebrafish larvae exposed to PTZ

**FIGURE 4:**
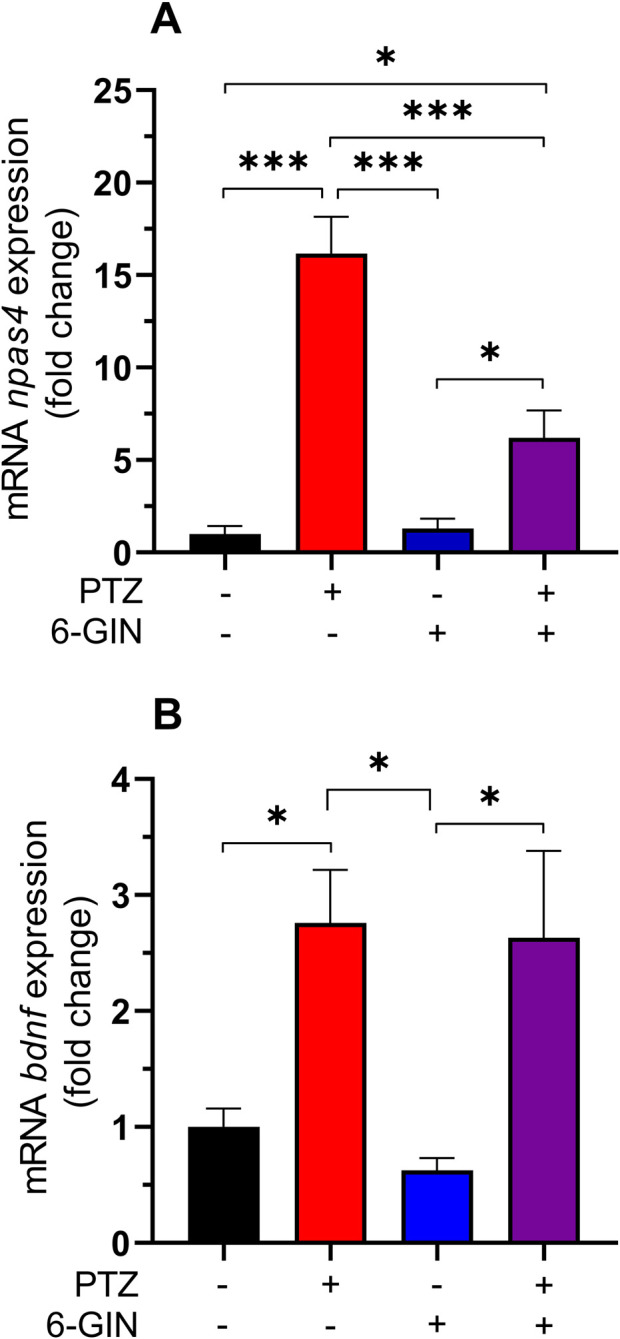
Effect of 6-gingerol on *npas4* and *bdnf* mRNA expression in the PTZ-treated zebrafish larvae. After a 22 h incubation in 6-gingerol (37.5 μM), zebrafish larvae were exposed to PTZ (20 mM) for 60 min. Next, zebrafish larvae were collected in a pool of n = 10/sample. mRNA levels were normalized against *18S and b2m.* Data were analyzed using one–way ANOVA with Tukey’s *post hoc* test: **(A)**, F(3, 19) = 32.12, p < 0.0001; **(B)**, F(3, 18) = 6.403, p = 0.0038. Data are depicted as a mean + SEM (n = 5–6/group). ***p < 0.001, *p < 0.05. 6-GIN, 6-gingerol; PTZ, pentylenetetrazole.

Expression of neuronal Per-Arnt-Sim (PAS) domain protein (*npas4*) and brain-derived neurotrophic factor (*bdnf*) on mRNA level was analyzed to verify behavioral effects noted in our previous studies in the PTZ-induced seizure test in zebrafish larvae (Gawel et al., 2021). Statistical analysis revealed that exposition to PTZ significantly increased both *npas4* (p < 0.001) and *bdnf* (p < 0.05) mRNA expression in zebrafish larvae. Although 6-gingerol at the concentration of 37.5 μM did not change expression of the studied genes in the control (vehicle-treated) group (p > 0.05), it significantly decreased *npas4* expression in the PTZ-exposed larvae (p < 0.001). mRNA expression of *bdnf* in group co-treated with PTZ and 6-gingerol did not differ significantly from the group exposed to PTZ (p > 0.05) and was significantly higher than in group treated with 6-gingerol. Results are presented in Figure 4 [one way ANOVA: *npas4* mRNA, F(3, 19) = 32.12, p < 0.0001; *bdnf* mRNA, F(3,18) = 6.403, p = 0.0038, with Tukey’s *post hoc* test].

### Effect of 6-gingerol on number of LFPs in *cacna1aa-* and *scn1lab-*morphants

**FIGURE 5:**
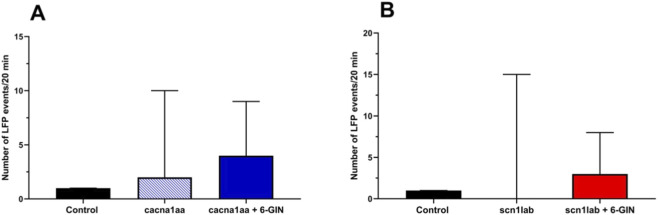
Effect of 6-gingerol on number of LFPs from the *optic tectum* of *cacna1aa* and *scn1lab* morphants. Zebrafish larvae were incubated with 6-gingerol (37.5 µM) or vehicle for 22 h and subsequently LFPs were registered during 20 min, at 4 dpf zebrafish larvae. The control group was the same in both experiments. Data are presented as number of epileptiform-like events (median + 95% confidence limits). Results were analyzed using Kruskal–Wallis test: **(A)**, KW = 3.546, p = 0.1699; **(B)**, KW = 1.388, p = 0.4995. 6-GIN - 6-gingerol.

In our study, we used a zebrafish model of Dravet syndrome and absence seizure using MOs targeting *scn1lab* and *cacna1aa* mRNA, respectively. Knockdown of both genes in zebrafish larvae resulted in abnormal brain activity characterized by the occurrence of discharges with amplitudes exceeding 3-times the background noise. Incubation with 6-gingerol (37.5 µM) for 22 h did not significantly affect the number of LFPs registered in the *optic tectum* of either *cacna1aa* (KW = 3.546, p = 0.1699) or *scn1lab* (KW = 1.388, p = 0.4995) morphants. Results are presented in Figure 5.

### Pharmacokinetic studies in mice

**FIGURE 6:**
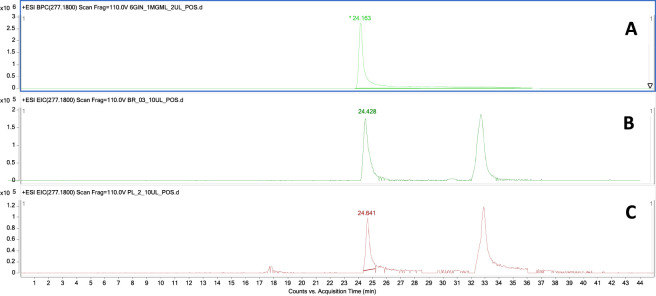
Extracted ion chromatograms of 6-gingerol from representative injections of a standard solution **(A)**, brain homogenate **(B)** and serum sample **(C)** under the applied chromatographic conditions with 6-gingerol eluted from the column at the 24th minute.

The developed LC-MS bioanalytical method was used for the determination of 6-gingerol in the brain and serum samples as shown in Figure 6. 6-Gingerol was eluted from the column around 24 min into the analysis. The recorded injections enabled the acquisition of peak areas corresponding to 6-gingerol in all analyzed samples providing data for the pharmacokinetic calculations.

Pharmacokinetic (PK) parameters calculated using non-compartmental analysis based on the concentration-time data after single i.p. administration of 6-gingerol at a dose of 10 mg/kg are presented in Table 2 and Figure 6. The maximal concentration in serum (as well as in the brain) was observed at the first sampling point, i.e., 15 min after administration, indicating rapid absorption. The elimination half-life was determined to be 1.73 h which for mice is relatively long. The percentage of water in fat-free wet weight for most mature animals is estimated between 70% and 76%, while some studies indicate that this figure is approximately 80% for mice. Thus, the volume of distribution (V_d_) of 6-gingerol determined in this study (758.08 mL/kg) indicates rather low distribution to organs and tissues and limited degree of tissue binding. The total clearance (CL) is much lower than the mouse liver blood flow (ca. 60–80 mL/min/kg) which might suggest that 6-gingerol is not extensively metabolized in the liver, however the values of V_d_ and CL calculated after i.p. administration is determined by F (fraction of dose absorbed) therefore these results should be taken with caution. Because pharmacokinetic parameters were estimated after i.p. dosing, CL/F and Vz/F depend on unknown bioavailability. The investigated compound is able to penetrate blood-brain barrier as the brain-to-serum ratio at 1 h after administration is 0.18. A higher brain to serum ratio (1.25) observed 6 h after administration indicates that 6-gingerol is eliminated from the brain much more slowly than from the blood, suggesting prolonged retention within brain tissue. Values of brain to serum ratio calculated at different time points are presented in Table 3. The chromatographic method used for the quantitative determinations was evaluated for the linearity range of 6-gingerol determinations, its LOD and LOQ values, inter- and intra-day stability. The calculated optimization parameters are presented in the Supplementary Material.

**TABLE 2:** Estimated pharmacokinetic parameters (non-compartmental analysis) of 6-gingerol calculated from the mean mice serum concentration values (n = 5) after its single i.p. administration at a dose of 10 mg/kg in Crl:CD1(ICR) mice.

Parameter	6-gingerol
C_max_ [µg/mL]	78.73
t_max_ [h]	0.25
λ_z_ [h^-1^]	0.4
t_0.5λz_ [h]	1.73
CL/F [mL/h/kg]	303.67
AUC_0-inf_ [µg·h/mL]	32.93
V_z_/F [mL/kg]	758.08
MRT [h]	0.9

C_max_, maximum concentration; t_max_, the time to reach C_max_; λz, terminal slope; t_0.5λz_, terminal half-life; CL, clearance; AUC_0-inf_, area under the mean serum concentration *versus* time curve extrapolated to infinity; V_z_, volume of distribution; MRT, mean residence time; F, fraction of dose absorbed.

**TABLE 3:** Brain to serum ratio for 6-gingerol calculated at different time points following its single i.p. administration at a dose of 10 mg/kg in Crl:CD1(ICR) mice.

Time [min]	Brain to serum ratio
15	8.61E-03
30	3.34E-02
45	1.11E-01
60	1.85E-01
120	2.94E-01
360	1.25E+00

### Acute seizure tests in mice

#### Electrically-induced seizure tests

In the 6 Hz psychomotor seizure test, 6-gingerol (60 and 90 mg/kg) did not show any significant effect when animals were stimulated with the current of 44 mA. In contrast, a statistically significant dose-dependent anticonvulsant effect of 6-gingerol was observed at an electrical stimulation intensity of 32 mA. In this case, 6-gingerol at a dose of 30 mg/kg protected 33.3% of the animals in the group (p = 0.1429), while administration of the studied compound at a dose of 60 mg/kg prevented psychomotor seizures in 72.7% of the animals (p = 0.014, Table 4).

**TABLE 4:** Effect of 6-gingerol on the occurrence of seizures in the MES and 6 Hz-induced psychomotor seizures tests in mice.

Seizure test	Treatment	Inhibition of seizures (%)	Number of animals in group	Statistical analysis
6 Hz test 32 mA	Control (1% Tween)	0%	9	–
	6-gingerol 30 mg/kg	33.3%	6	p = 0.1429
	6-gingerol 60 mg/kg	72.7%	11	p = 0.014
6 Hz test 44 mA	Control (1% Tween)	0%	8	–
	6-gingerol 60 mg/kg	0%	8	p = 1.0
	6-gingerol 90 mg/kg	0%	8	p = 1.0
MES	Control (1% Tween)	0%	9	–
	6-gingerol 10 mg/kg	0%	6	p = 1.0
	6-gingerol 60 mg/kg	0%	11	p = 1.0

Data were analysed using Fisher’s exact test.

6-gingerol administered i.p. at doses of 10 and 60 mg/kg 15 min before the MES test did not protect mice from the tonic hind limb extension induced by electrical stimulation at an intensity of 50 mA (p = 1.0). Results are presented in Table 4.

### The i.v. PTZ seizure threshold test

**FIGURE 7:**
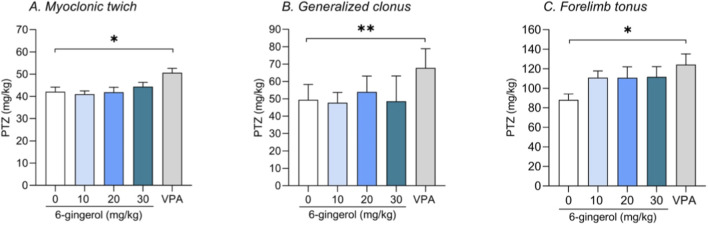
Effect of 6-gingerol on threshold for the myoclonic twitches **(A)**, generalized clonus **(B)** and forelimb tonus **(C)** in the iv. PTZ test in mice. 6-gingerol (10–30mg/kg) and VPA (150 mg/kg, as a positive control) were administered ip. 15 and 30 min before the test, respectively. The negative control group was treated with 1% Tween solution in saline. Experimental groups consisted of 12–18 mice. Data on myoclonic twitches **(A)** and forelimb tonus seizures **(C)** are presented as mean dose of PTZ (in mg/kg) that provoke the respective kind of seizures + SEM and were analysed using one-way ANOVA with Dunnett’s *post hoc* (myoclonic twitch, F(4, 72) = 4.569, p = 0.0024; forelimb tonus, F(4, 37) = 2.007, p = 0.1024). Data on generalized clonic seizures are presented as median + 95% conficence limits and were analysed using Kruskal–Wallis test followed with Dunn’s *post hoc* test (generalized clonus, KW = 19.97, p = 0.0005). *p ≤ 0.05 and **p ≤ 0.01. PTZ, pentylenetetrazole; VPA, sodium valproate.

Figure 7 presents the effect of 6-gingerol (i.p., 10–30 mg/kg) on the seizure threshold for three types of seizures, i.e., the myoclonic twitches, generalized clonus with loss of right reflex and tonic hindlimb extension, in the i.v. PTZ test in mice. Although one-way ANOVA and Kruskal–Wallis test indicated a significant group effect (one-way ANOVA: myoclonic twitch, F(4, 72) = 4.569, p = 0.0024; forelimb tonus, F(4, 37) = 2.007, p = 0.1024; Kruskal–Wallis test: generalized clonus, KW = 19.97, p = 0.0005), *post hoc* analysis revealed that this was driven exclusively by the VPA-treated group. The only statistically significant anticonvulsant effects were observed in the experimental group treated with VPA at a dose of 150 mg/kg.

### Effect of 6-gingerol on the motor coordination and muscle strength in mice

**FIGURE 8:**
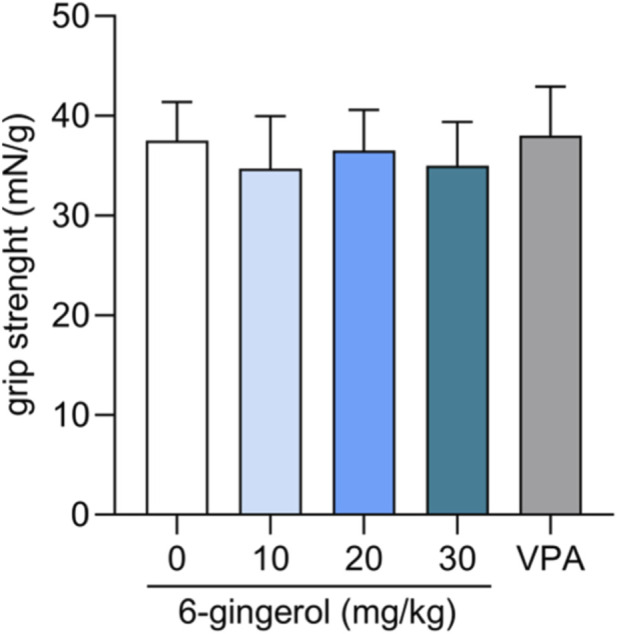
Effect of 6-gingerol on the neuromuscular strength in mice. 6-gingerol (10–30 mg/kg) and VPA (150 mg/kg, as a positive control) were administered i.p. 15 and 30 min before the test, respectively. Negative control group was treated with 1% Tween solution in saline. Experimental groups consisted of 16 mice. Results are presented as the mean (+ SEM) grip strengths in milinewtons per gram of mouse body weight (mN/g) and were analysed with one-way ANOVA (F (4, 75) = 1.689, p = 0.1615).

Acute i.p. treatment with 6-gingerol at doses ranging from 30 to 90 mg/kg did not affect motor coordination in mice as assessed using the rotarod test (p > 0.05) (data not shown). Additionally, no significant impairment of motor coordination was observed in the chimney test following administration of the studied compound at doses of 10–30 mg/kg (one-way ANOVA: F (4, 75) = 1.689, p = 0.1615) (Figure 8).

## Discussion

6-gingerol is the most abundant active compound found in the ginger rhizome extracts. Consequently, it is also the most widely studied compound for potential therapeutic properties. Studies conducted several years ago demonstrated that ginger extracts have anticonvulsant properties in the PTZ-induced mouse experimental seizure models (Hosseini and Mirazi, 2014; 2015; Hosseini et al., 2016) and prompted further research aimed to more precisely determine properties of the extracts in other experimental models of seizures and, in particular, identify the active compounds responsible for anticonvulsant effect. In the previous study, we demonstrated the anticonvulsant effect of 6-gingerol in the zebrafish larvae PTZ-induced seizure assay, which manifested as a decrease in PTZ-induced hyperlocomotion and reduction in the number and mean duration of LFP events recorded in the *optic tectum* of the PTZ-exposed larvae. This effect was additionally associated with a reduction in glutamate level and glutamate/GABA ratio, as well as a decrease in mRNA expression of *grin2b*–gene encoding a subunit of NMDA (N-methyl-D-aspartate)-type glutamate ionotropic receptor (Gawel et al., 2021). We are now following up on this initial study using other zebrafish larvae seizure models (i.e., EKP- and PILO-induced seizure tests and *scn1lab* and *cacna1aa* morphants) as well as some commonly used seizure models in mice (i.e., electrically-induced seizures–MES and 6 Hz psychomotor seizure tests, and the i.v. PTZ seizure test). Pharmacokinetic parameters of 6-gingerol in mice, as well as its ability to cross the blood-brain barrier after i.p. administration, were also assessed.

In our present study, we evaluated changes in *npas4* and *bdnf* mRNA expression in PTZ-exposed zebrafish larvae pre-incubated with 6-gingerol to additionally confirm the anticonvulsant effect of this compound noted in our previous study (Gawel et al., 2021). Both of these factors are used in preclinical studies as significant markers of neuronal activity (Sun and Lin, 2016). *Npas4* is one of the recently described immediate early genes (IEGs) that is expressed only in neurons. Its activation takes place after recent neuronal activity and is mediated by intracellular free calcium as a second messenger. The results of preclinical studies conducted using experimental models of seizures and epilepsy indicate that NPAS4 protein performs a protective function in the brain by restoring homeostasis of neurons activity. It was noted that PTZ-induced convulsant activity upregulated *npas4* expression in the mouse hippocampus and, moreover, inactivation of this gene facilitates PTZ kindling development (Shan et al., 2018). In PILO-induced epileptic rats, the level of NPAS4 protein increased during the acute phase of seizures (6–72 h) and decreased during the chronic phase (7–60 days). In PILO-treated rats with silenced *Npas4*, convulsions occurred more frequently and were longer (Wang et al., 2014). In PILO-treated zebrafish larvae, *npas4* was overexpressed 22 h after cessation of treatment (Gawel et al., 2024). NPAS4 directly controls the expression of numerous activity-dependent genes, i.e., *bdnf* and *homer1a*, and acts, among others, as a regulator of homeostasis in inhibitory and excitatory neurotransmission processes (Fu et al., 2020; Sun and Lin, 2016; Shan et al., 2018). Disruption of the excitatory-inhibitory balance could lead to neuronal death and impairment of the nervous system function, including the development of epilepsy. BDNF is a neurotrophin family member that controls, among others, neuronal survival and development, long-term potentiation (LTP) and plasticity processes in the brain, and therefore, is strictly connected to seizures and epilepsy development (AlRuwaili et al., 2024; Gliwińska et al., 2023). Our results on *npas4* and *bdnf* mRNA expression follow the previous findings which showed increased expression of these markers in the brains of experimental animals after exposure to PTZ (Abdelaziz et al., 2025; Seo et al., 2020; Lopes et al., 2016; Klarić et al., 2014; Torres-Hernández et al., 2016; Shan et al., 2018). Moreover, some compounds that showed anticonvulsant properties in experimental seizure and epilepsy models normalise the expression of these markers (Torres-Hernández et al., 2016; Seo et al., 2020; Abdelaziz et al., 2025). Although 6-gingerol did not significantly affect expression of *bdnf* mRNA in the PTZ-exposed zebrafish larvae, it significantly reduced *npas4* mRNA expression, which supports its anticonvulsant activity. Similar effects were noted in the case of another plant-derived compound with anticonvulsant effect–D-limonene–terpene found in citrus fruits (Seo et al., 2020). Although the involvement of *naps4* and *bdnf* genes in seizure processes is clear, the current state of knowledge does not allow for an unambiguous determination of their role in the anticonvulsant mechanism of action of 6-gingerol in PTZ-induced seizure models. This issue requires further research.

Apart from the continuation of the study on the effect of 6-gingerol in PTZ-treated zebrafish larvae, we also evaluated the activity of this compound in two other chemoconvulsant-induced seizure models in zebrafish larvae, i.e., in the EKP- and PILO-induced seizure tests. EKP-induced seizures are described as a model of pharmacoresistant seizures (Zhang et al., 2017). We noted that 6-gingerol (37.5 μM) significantly weakened EKP-induced seizure activity but did not affect seizures induced after PILO exposure. Proconvulsant activity of EKP results from inhibition of glutamic acid decarboxylase, which transforms glutamic acid into GABA (Zhang et al., 2017). Similar to PTZ, the action of EKP is closely linked to the attenuation of GABAergic neurotransmission. Results from the PTZ- and EKP-induced seizure tests suggest that 6-gingerol could exert its anticonvulsant effect by enhancement of the GABAergic system and is not able to counteract seizures induced by enhancement of cholinergic neurotransmission. PILO acts as a cholinomimetic drug that stimulates muscarinic receptors. In rodents, administration of PILO induces limbic seizures which progress to status epilepticus (Turski et al., 1984). In zebrafish larvae, acute administration of PILO results in behavioural inhibition. At the EEG level, numerous high-amplitude discharges are observed (Gawel et al., 2024). Since, in our hands, 6-gingerol had no effect on the behaviour of the larvae in the PILO-induced seizure test, it seems unlikely that it could be effective in the treatment of temporal lobe epilepsy in humans.

In this study, we next used two zebrafish larvae models of seizures induced by knocking down genes important in the proper activity of the brain and epileptogenesis process. *Scn1lab* encodes voltage-gated sodium channel alpha subunit Na_v_1.1 and *scn1lab* morphants are described as zebrafish model of Dravet syndrome–one of the most devastating and pharmacoresistant types of childhood epilepsy syndromes (Zhang et al., 2015; Baraban et al., 2013). Children with Dravet syndrome experience a variety of seizures i.g. tonic-clonic, clonic, myoclonic, atypical absence seizures, atonic seizures, status epilepticus (Li et al., 2021; Selvarajah et al., 2021). The substances currently approved as add-on therapy for the treatment of seizures in Dravet syndrome are fenfluramine (serotonin-enhancing agent), cannabidiol (ion channel modulator, effects on the endocannabinoid or adenosine systems), and stiripentol (among others positive allosteric modulator of GABA_A_ receptors) (Guerrini et al., 2024). Tiraboschi et al. (2020) were the first to demonstrate the mechanism of antiepileptic properties of fenfluramine in *scn1lab* ^*−/−*^ mutants. Using zebrafish *scn1lab* ^*−/−*^ mutants, Baraban et al. (2013) pinpointed clemizole (antihistamine agent) being now under phase III clinical trials for Dravet syndrome patients (https://clinicaltrials.gov/study/NCT04462770). Loss of function of *cacna1aa* gene results in disturbances in P/Q type calcium channel activity, which consequently might lead to seizures (Gawel et al., 2020). In zebrafish *cacna1aa* morphants, EEG discharges were observed (Gawel et al., 2020). The administration of ASMs registered for the treatment of absence seizures in humans reduced the number of discharges in the EEG (ethosuximide, valproic acid, lamotrigine and topiramate-note that a common feature of these drugs is their effect on the calcium ion channels). Although 6-gingerol effectively blocked seizures induced by exposure to EKP, it was ineffective in preventing seizures in Dravet syndrome model as well as seizures in *cacna1aa* morphants. The lack of anticonvulsant effect in these models might result from the fact that epileptiform activity in the morphants is mediated by disturbances in the activity of sodium and calcium channels, while our results from the PTZ- and EKP-induced seizure tests suggest that 6-gingerol mechanism of anticonvulsant action is mainly GABAergic.

Although 6-gingerol inhibits both kinds of seizures induced by chemoconvulsants that reduce GABAergic neurotransmission in zebrafish larvae, it did not show an anticonvulsant effect in the timed infusion PTZ seizure test in mice. A requirement for all drugs affecting central nervous system functions, including seizure activity, is the ability to cross the blood-brain barrier. According to the data available in the scientific literature and our results, 6-gingerol is able to pass the blood-brain barrier (Simon et al., 2020; Lim et al., 2025; Kuswandani et al., 2025). Pharmacokinetic study revealed that 6-gingerol reaches its maximum concentration in brain tissue as early as 15 min after its i.p. injection, therefore, its anticonvulsant activity in mouse seizure tests was assessed at this time point. The lack of the anticonvulsant effect of 6-gingerol in the i.v. PTZ seizure threshold test might be because, despite its ability to cross the blood-brain barrier, its concentration in brain tissue after i.p. administration at a dose of 30 mg/kg was insufficient to affect the targets relevant to anticonvulsant activity.

In our study, we also employed two mouse models of seizures induced by electrical stimulation, namely, the 6 Hz-induced psychomotor seizure and MES tests. Psychomotor seizures are evoked by a low-frequency (i.e. 6 Hz), long-duration (i.e., 3 s) electrical stimulus with rectangular pulses of 0.2 ms duration and manifested as twitching vibrissae, stun position, head nodding, jaw movements, limb clonus and Straub’s tail. Two intensities of stimulation are typically used in this test, i.e., 32 or 44 mA. The 6 Hz psychomotor seizure test with higher intensity of stimulation (i.e. 44 mA) might be considered a model of pharmacoresistant seizures since this kind of seizures are not alleviated by a majority of ASMs (Löscher and White, 2023; Barton et al., 2001). We noted a significant anticonvulsant effect of 6-gingerol (60 mg/kg) in the psychomotor seizure test, but only in the low-intensity stimulation condition, which is not a model of pharmacoresistant seizures. The specific mechanism of action of anticonvulsants inhibiting psychomotor seizures is not clearly defined, however, many ASMs that effectively inhibit the 6 Hz-induced psychomotor seizures act by modulating GABAergic neurotransmission, i.e., clonazepam, phenobarbital and tiagabine (Löscher and White, 2023).

It should be noted here that in the psychomotor seizure and MES tests, the range of 6-gingerol doses tested was higher than in the i.v. PTZ test. This discrepancy was due to the fact that these tests were performed on two different strains of mice, i.e., CD-1 and Crl:CD1(ICR), which could have affected the animals’ sensitivity to 6-gingerol. Doses above 30 mg/kg caused adverse peripheral effects in Crl:CD1(ICR) mice, including immobility and problems with maintaining proper body posture, while in CD-1 mice, these doses were safe and did not cause significant impairment of motor coordination, which was assessed in the rotarod test. The genetic background of mice might affect the bioavailability and pharmacokinetics of 6-gingerol after i.p. administration, which influences its effectiveness. Strain-dependent changes in pharmacokinetic parameters have been observed previously for cocaine (McCarthy et al., 2004; Zhu et al., 2021). Epigenetic and environmental factors are also responsible for variability in drug response (Löscher, 2024). Anticonvulsant effect noted in the 6 Hz psychomotor seizure test was caused by a high dose of 6-gingerol, i.e., 60 mg/kg. In the i.v. PTZ test, the highest tested dose of this compound was 30 mg/kg. Administration of a higher dose of gingerol in the 6 Hz test might have increased brain concentration of this compound to a level sufficient to affect the targets responsible for anticonvulsant action. Therefore, higher concentrations of the tested compound led to a significant anticonvulsant effect in the 6 Hz test, which was not observed with lower doses in the i.v. PTZ test.

The MES test is considered a model of generalised tonic-clonic seizures in humans. Brief (i.e., 0.2 s), high-intensity (i.e., 50 mA) and high-frequency (i.e., 50 Hz) electrical stimulation provokes maximal seizures characterised by forelimb tonic extension followed by clonic convulsions of both forelimbs and hindlimbs. In this test, anticonvulsant effect is demonstrated by drugs that act mainly by sodium channels inhibition, i.e., carbamazepine, lamotrigine and topiramate (Castel-Branco et al., 2009; Löscher et al., 1991; White et al., 1995). 6-gingerol did not reduce the occurrence of seizures in the MES test in mice.

Although we used three different models of epileptic seizures in our study, differing in the mechanism of their induction, we did not use any chronic epilepsy model in rodents that would allow us to evaluate the anti-epileptic effect of 6-gingerol. Another significant limitation of our study is the lack of detailed pharmacokinetic studies that would cover at least a 24-h period after administration of 6-gingerol. That would enable a more accurate assessment of this compound’s biodistribution in animal organisms. Our research has not yet clarified the molecular mechanisms underlying the anticonvulsant effects of 6-gingerol, and should be considered as a preliminary step towards further investigation into the potential use of 6-gingerol in the treatment of epilepsy. Although the experiments conducted suggest a GABAergic mechanism of action, they do not allow for the precise determination of the targets for 6-gingerol or the types of seizures that could be inhibited/limited by this compound. To do this, further research is needed, including looking at modifying the form of 6-gingerol administration (i.e., nanoparticles) to increase its penetration into the brain.

Also, should this research progress, a comprehensive standardization protocol would be imperative. This would involve establishing a fully characterized primary reference standard for 6-gingerol and validating a quantitative analytical method, such as the described HPLC-based assay, in accordance with ICH Q2(R1) guidelines to ensure its specificity, linearity, accuracy, and robustness. This validated assay would then be fundamental for conducting stability studies in accordance with ICH Q1A(R2) guidelines to determine the compound’s shelf-life and optimal storage conditions. These procedures, however, should be developed in the future as more in-depth investigations.

It is worth noting that our findings are related to the single isolated phytochemical, 6-gingerol, and should not be extrapolated to the crude ginger extract. The use of whole herbal extracts as therapeutic agents presents formidable challenges for standardization due to their inherent chemical complexity and the significant, often uncontrolled, variability in constituent profiles. This variability stems from diverse factors, including plant genetics, geographical origin, harvesting time, and processing methods. To ensure dose-to-dose consistency, predictable pharmacokinetics, and a well-defined mechanism of action—important prerequisites for a modern therapeutic agent—the future development must focus on a single, purified chemical component. Therefore, 6-gingerol, rather than a chemically characterized extract, represents the only viable path forward for potential clinical development.

## Conclusion

Our studies revealed some anticonvulsant properties of 6-gingerol, which were possibly mediated by the enhancement of GABAergic neurotransmission. Anticonvulsant effect was noted in the case of seizures induced by chemoconvulsants affecting GABAergic neurotransmission in zebrafish larvae and the 6 Hz psychomotor seizure test (32 mA) in mice when a high dose of the compound was used. The limited anticonvulsant effect of 6-gingerol might be related to its insufficient penetration into brain tissue. To achieve a stronger and significant anticonvulsant effect, the use of appropriate nanoparticles as carriers of 6-gingerol should be considered, which would enable its easier penetration through the blood-brain barrier and thus increase its concentration in brain tissue. Although 6-gingerol is the primary ingredient of extracts from ginger, there are also several other phenolic and terpene compounds, i.e., 8- and 10-gingerol, shogaols, zingerone, as well as paradols. The significant anticonvulsant effect of extracts from ginger roots noted in the previous studies might result from the synergistic activity of different compounds included in the studied extracts. In summary, further research is needed to fully assess the anticonvulsant potential of 6-gingerol and its mechanisms of action.

## Data Availability

The original contributions presented in the study are included in the article/Supplementary Material, further inquiries can be directed to the corresponding author.

## References

[B1] Abdelaziz H. A. Hamed M. F. Ghoniem H. A. Nader M. A. Suddek G. M. (2025). Empagliflozin mitigates ptz-induced seizures in rats: Modulating npas4 and creb-bdnf signaling pathway. J. Neuroimmune Pharmacol. 20 (1), 5. 10.1007/s11481-024-10162-6 39776284 PMC11706855

[B2] AlRuwaili R. Al-kuraishy H. M. Al-Gareeb A. I. Ali N. H. Alexiou A. Papadakis M. (2024). The possible role of brain-derived neurotrophic factor in epilepsy. Neurochem. Res. 49 (3), 533–547. 10.1007/s11064-023-04064-x 38006577 PMC10884085

[B3] Baraban S. C. Dinday M. T. Hortopan G. A. (2013). Drug screening in scn1a zebrafish mutant identifies clemizole as a potential Dravet syndrome treatment. Nat. Commun. 4, 2410. 10.1038/ncomms3410 24002024 PMC3891590

[B4] Barton M. E. Klein B. D. Wolf H. H. Steve W. H. (2001). Pharmacological characterization of the 6 hz psychomotor seizure model of partial epilepsy. Epilepsy Res. 47 (3), 217–227. 10.1016/s0920-1211(01)00302-3 11738929

[B5] Belete T. M. (2023). Recent progress in the development of new antiepileptic drugs with novel targets. Ann. Neurosci. 30 (4), 262–276. 10.1177/09727531231185991 38020406 PMC10662271

[B6] Blanco A. M. Bertucci J. I. Hatef A. Unniappan S. (2020). Feeding and food availability modulate brain-derived neurotrophic factor, an orexigen with metabolic roles in zebrafish. Sci. Rep. 10 (1), 10727. 10.1038/s41598-020-67535-z 32612127 PMC7329848

[B7] Boissier J. R. Tardy J. Diverres J. C. (2008). Une nouvelle méthode simple pour explorer l’action «tranquillisante»: le test de la cheminée. Med. Exp. 3 (1), 81–84. 10.1159/000134913

[B8] Borowicz-Reutt K. Czernia J. Krawczyk M. (2024). Cbd in the treatment of epilepsy. Molecules 29 (9), 1981. 10.3390/molecules29091981 38731471 PMC11085483

[B9] Castel-Branco M. M. Alves G. L. Figueiredo I. V. Falcão A. C. Caramona M. M. (2009). The maximal electroshock seizure (mes) model in the preclinical assessment of potential new antiepileptic drugs. Methods Find. Exp. Clin. Pharmacol. 31 (2), 101–106. 10.1358/mf.2009.31.2.1338414 19455265

[B10] Damar U. Gersner R. Johnstone J. T. Schachter S. Rotenberg A. (2016). Huperzine a as a neuroprotective and antiepileptic drug: a review of preclinical research. Expert Rev. Neurother. 16 (6), 671–680. 10.1080/14737175.2016.1175303 27086593

[B11] Dunham N. W. Miya T. S. (1957). A note on a simple apparatus for detecting neurological deficit in rats and mice. J. Am. Pharm. Assoc. Am. Pharm. Assoc. 46 (3), 208–209. 10.1002/jps.3030460322 13502156

[B12] Fu J. Guo O. Zhen Z. Zhen J. (2020). Essential functions of the transcription factor npas4 in neural circuit development, plasticity, and diseases. Front. Neurosci., 14–2020. 10.3389/fnins.2020.603373 33335473 PMC7736240

[B13] Gawel K. Turski W. A. van der Ent W. Mathai B. J. Kirstein-Smardzewska K. J. Simonsen A. (2020). Phenotypic characterization of larval zebrafish (*Danio rerio*) with partial knockdown of the cacna1a gene. Mol. Neurobiol. 57 (4), 1904–1916. 10.1007/s12035-019-01860-x 31875924 PMC7118054

[B14] Gawel K. Kukula-Koch W. Banono N. S. Nieoczym D. Targowska-Duda K. M. Czernicka L. (2021). 6-gingerol, a major constituent of *Zingiber officinale* rhizoma, exerts anticonvulsant activity in the pentylenetetrazole-induced seizure model in larval zebrafish. Int. J. Mol. Sci. 22 (14), 7745. 10.3390/ijms22147745 34299361 PMC8305044

[B15] Gawel K. Hulas-Stasiak M. Marszalek-Grabska M. Grenda A. Siekierska A. Kosheva N. (2024). Induction of seizures and initiation of epileptogenesis by pilocarpine in zebrafish larvae. Front. Mol. Neurosci. 17, 1418606. 10.3389/fnmol.2024.1418606 39165716 PMC11333333

[B16] Gliwińska A. Czubilińska-Łada J. Więckiewicz G. Świętochowska E. Badeński A. Dworak M. (2023). The role of brain-derived neurotrophic factor (bdnf) in diagnosis and treatment of epilepsy, depression, schizophrenia, anorexia nervosa and alzheimer’s disease as highly drug-resistant diseases: a narrative review. Brain Sci. 13 (2), 163. 10.3390/brainsci13020163 36831706 PMC9953867

[B17] Guerrini R. Chiron C. Vandame D. Linley W. Toward T. (2024). Comparative efficacy and safety of stiripentol, cannabidiol and fenfluramine as first-line add-on therapies for seizures in Dravet syndrome: a network meta-analysis. Epilepsia Open 9 (2), 689–703. 10.1002/epi4.12923 38427284 PMC10984299

[B18] Hosseini A. Mirazi N. (2014). Acute administration of ginger (*Zingiber officinale* rhizomes) extract on timed intravenous pentylenetetrazol infusion seizure model in mice. Epilepsy Res. 108 (3), 411–419. 10.1016/j.eplepsyres.2014.01.008 24529324

[B19] Hosseini A. Mirazi N. (2015). Alteration of pentylenetetrazole-induced seizure threshold by chronic administration of ginger (*Zingiber officinale*) extract in male mice. Pharm. Biol. 53 (5), 752–757. 10.3109/13880209.2014.942789 25609148

[B20] Hosseini A. Mirazi N. Gomar A. (2016). Protective effect of ginger against the pentylenetetrazole-induced seizure threshold model in streptozocin treated-diabetic mice. Physiology Pharmacol. 20, 108–116.

[B21] Klarić T. Lardelli M. Key B. Koblar S. Lewis M. (2014). Activity-dependent expression of neuronal pas domain-containing protein 4 (npas4a) in the developing zebrafish brain. Front. Neuroanat. 8, 148. 10.3389/fnana.2014.00148 25538572 PMC4255624

[B22] Kuswandani F. Wilar G. Wahyuni I. S. Megantara S. Pitaloka D. A. E. Levita J. (2025). Gingerols and shogaols of *Zingiber officinale* var. Sunti valeton as potential allosteric agonists of human gaba(a) receptor by *in silico* pharmacology approach. J. Exp. Pharmacol. 17, 359–374. 10.2147/JEP.S524890 40547511 PMC12182062

[B23] Li W. Schneider A. L. Scheffer I. E. (2021). Defining Dravet syndrome: an essential pre-requisite for precision medicine trials. Epilepsia 62 (9), 2205–2217. 10.1111/epi.17015 34338318 PMC9291974

[B24] Lim S. W. Chen W. C. Ko H. J. Su Y. F. Wu C. H. Huang F. L. (2025). 6-gingerol induced apoptosis and cell cycle arrest in glioma cells *via* mnsod and erk phosphorylation modulation. Biomol. Ther. Seoul. 33 (1), 129–142. 10.4062/biomolther.2024.084 39632791 PMC11704400

[B25] Lopes M. W. Sapio M. R. Leal R. B. Fricker L. D. (2016). Knockdown of carboxypeptidase a6 in zebrafish larvae reduces response to seizure-inducing drugs and causes changes in the level of mrnas encoding signaling molecules. PLoS One 11 (4), e0152905. 10.1371/journal.pone.0152905 27050163 PMC4822968

[B26] Löscher W. (2024). Of mice and men: the inter-individual variability of the brain's response to drugs. eNeuro 11 (2). 10.1523/ENEURO.0518-23.2024 38355298 PMC10867552

[B27] Löscher W. White H. S. (2023). Animal models of drug-resistant epilepsy as tools for deciphering the cellular and molecular mechanisms of pharmacoresistance and discovering more effective treatments. Cells 12 (9), 1233. 10.3390/cells12091233 37174633 PMC10177106

[B28] Löscher W. Fassbender C. P. Nolting B. (1991). The role of technical, biological and pharmacological factors in the laboratory evaluation of anticonvulsant drugs. Ii. Maximal electroshock seizure models. Epilepsy Res. 8 (2), 79–94. 10.1016/0920-1211(91)90075-q 2065646

[B29] Löscher W. Klitgaard H. Twyman R. E. Schmidt D. (2013). New avenues for anti-epileptic drug discovery and development. Nat. Rev. Drug Discov. 12 (10), 757–776. 10.1038/nrd4126 24052047

[B30] Löscher W. Potschka H. Sisodiya S. M. Vezzani A. (2020). Drug resistance in epilepsy: clinical impact, potential mechanisms, and new innovative treatment options. Pharmacol. Rev. 72 (3), 606–638. 10.1124/pr.120.019539 32540959 PMC7300324

[B31] Łukasiuk K. Lasoń W. (2023). Emerging molecular targets for anti-epileptogenic and epilepsy modifying drugs. Int. J. Mol. Sci. 24 (3), 2928. 10.3390/ijms24032928 36769250 PMC9917847

[B32] Mandhane S. N. Aavula K. Rajamannar T. (2007). Timed pentylenetetrazol infusion test: a comparative analysis with s.C.Ptz and mes models of anticonvulsant screening in mice. Seizure 16 (7), 636–644. 10.1016/j.seizure.2007.05.005 17570689

[B33] Martimbianco A. L. C. Silva R. B. Cruz L. C. O. de Toledo I. P. Pacheco R. L. Colpani V. (2025). Cannabis derivatives and their synthetic analogs for treatment-resistant epilepsy: a systematic review and meta-analysis. Epilepsy Res. 214, 107559. 10.1016/j.eplepsyres.2025.107559 40267856

[B34] McCarthy L. E. Mannelli P. Niculescu M. Gingrich K. Unterwald E. M. Ehrlich M. E. (2004). The distribution of cocaine in mice differs by age and strain. Neurotoxicology Teratol. 26 (6), 839–848. 10.1016/j.ntt.2004.07.004 15451047

[B35] McCurley A. T. Callard G. V. (2008). Characterization of housekeeping genes in zebrafish: male-female differences and effects of tissue type, developmental stage and chemical treatment. BMC Mol. Biol. 9 (1), 102. 10.1186/1471-2199-9-102 19014500 PMC2588455

[B36] Meyer O. A. Tilson H. A. Byrd W. C. Riley M. T. (1979). A method for the routine assessment of fore- and hindlimb grip strength of rats and mice. Neurobehav. Toxicol. 1 (3), 233–236. 551317

[B37] Moreira F. A. de Oliveira A. C. P. Santos V. R. Moraes M. F. D. (2024). Cannabidiol and epilepsy. Int. Rev. Neurobiol. 177, 135–147. 10.1016/bs.irn.2024.03.009 39029983

[B38] Nieoczym D. Marszalek-Grabska M. Szalak R. Kundap U. Kaczor A. A. Wrobel T. M. (2024). A comprehensive assessment of palmatine as anticonvulsant agent - *in vivo* and *in silico* studies. Biomed. Pharmacother. 172, 116234. 10.1016/j.biopha.2024.116234 38325264

[B39] Rubio C. Romo-Parra H. López-Landa A. Rubio-Osornio M. (2024). Classification of current experimental models of epilepsy. Brain Sci. 14 (10), 1024. 10.3390/brainsci14101024 39452036 PMC11506208

[B40] Selvarajah A. Zulfiqar-Ali Q. Marques P. Rong M. Andrade D. M. (2021). A systematic review of adults with Dravet syndrome. Seizure - Eur. J. Epilepsy 87, 39–45. 10.1016/j.seizure.2021.02.025 33677403

[B41] Seo S. Song Y. Gu S. M. Min H. K. Hong J. T. Cha H. J. (2020). D-limonene inhibits pentylenetetrazole-induced seizure *via* adenosine a2a receptor modulation on gabaergic neuronal activity. Int. J. Mol. Sci. 21 (23), 9277. 10.3390/ijms21239277 33291789 PMC7730947

[B42] Shan W. Nagai T. Tanaka M. Itoh N. Furukawa-Hibi Y. Nabeshima T. (2018). Neuronal pas domain protein 4 (npas4) controls neuronal homeostasis in pentylenetetrazole-induced epilepsy through the induction of homer1a. J. Neurochem. 145 (1), 19–33. 10.1111/jnc.14274 29222951

[B43] Simon A. Darcsi A. Kery A. Riethmuller E. (2020). Blood-brain barrier permeability study of ginger constituents. J. Pharm. Biomed. Anal. 177, 112820. 10.1016/j.jpba.2019.112820 31476432

[B44] Sun X. Lin Y. (2016). Npas4: linking neuronal activity to memory. Trends Neurosci. 39 (4), 264–275. 10.1016/j.tins.2016.02.003 26987258 PMC4818711

[B45] Thijs R. D. Surges R. O'Brien T. J. Sander J. W. (2019). Epilepsy in adults. Lancet 393 (10172), 689–701. 10.1016/s0140-6736(18)32596-0 30686584

[B46] Tiraboschi E. Martina S. van der Ent W. Grzyb K. Gawel K. Cordero-Maldonado M. L. (2020). New insights into the early mechanisms of epileptogenesis in a zebrafish model of Dravet syndrome. Epilepsia 61 (3), 549–560. 10.1111/epi.16456 32096222

[B47] Torres-Hernández B. A. Colón L. R. Rosa-Falero C. Torrado A. Miscalichi N. Ortíz J. G. (2016). Reversal of pentylenetetrazole-altered swimming and neural activity-regulated gene expression in zebrafish larvae by valproic acid and valerian extract. Psychopharmacology 233 (13), 2533–2547. 10.1007/s00213-016-4304-z 27165438 PMC4908174

[B48] Turski W. A. Cavalheiro E. A. Bortolotto Z. A. Mello L. M. Schwarz M. Turski L. (1984). Seizures produced by pilocarpine in mice: a behavioral, electroencephalographic and morphological analysis. Brain Res. 321 (2), 237–253. 10.1016/0006-8993(84)90177-x 6498517

[B49] Wang D. Ren M. Guo J. Yang G. Long X. Hu R. (2014). The inhibitory effects of npas4 on seizures in pilocarpine-induced epileptic rats. PLoS One 9 (12), e115801. 10.1371/journal.pone.0115801 25536221 PMC4275263

[B50] White H. S. Johnson M. Wolf H. H. Kupferberg H. J. (1995). The early identification of anticonvulsant activity: role of the maximal electroshock and subcutaneous pentylenetetrazol seizure models. Ital. J. Neurol. Sci. 16 (1-2), 73–77. 10.1007/BF02229077 7642355

[B51] Woodbury L. A. Davenport V. D. (1952). Design and use of a new electroshock seizure apparatus, and analysis of factors altering seizure threshold and pattern. Arch. Int. Pharmacodyn. Ther. 92 (1), 97–107. 13008534

[B52] Zhang Y. Kecskés A. Copmans D. Langlois M. Crawford A. D. Ceulemans B. (2015). Pharmacological characterization of an antisense knockdown zebrafish model of Dravet syndrome: inhibition of epileptic seizures by the serotonin agonist fenfluramine. PLoS One 10 (5), e0125898. 10.1371/journal.pone.0125898 25965391 PMC4428833

[B53] Zhang Y. Vanmeert M. Siekierska A. Ny A. John J. Callewaert G. (2017). Inhibition of glutamate decarboxylase (gad) by ethyl ketopentenoate (ekp) induces treatment-resistant epileptic seizures in zebrafish. Sci. Rep. 7 (1), 7195. 10.1038/s41598-017-06294-w 28775328 PMC5543107

[B54] Zhu J. Beechinor R. J. Thompson T. Schorzman A. N. Zamboni W. Crona D. J. (2021). Pharmacokinetic and pharmacodynamic analyses of cocaine and its metabolites in behaviorally divergent inbred mouse strains. Genes Brain Behav. 20 (2), e12666. 10.1111/gbb.12666 32383297 PMC7941260

