# Supplementary material for: Studies on the anticonvulsant potential of 6-gingerol, an active ingredient of ginger rhizome
Source: Front Pharmacol. 2026 Apr 23;17:1740324. doi: 10.3389/fphar.2026.1740324 (PMC13149478; doi:10.3389/fphar.2026.1740324)
Supplement: Supplementary file 1 [file Supplementaryfile2.docx]

SUPPLEMENTARY FILE

**Isolation of 6-gingerol**

6-Gingerol was isolated from the ethanol extract obtained from the rhizomes of *Zingiber officinale.* Fresh rhizomes of Peruvian origin were purchased in the local market in Lublin, Poland, in May 2023. The rhizomes were finely sliced and dried for three days in the oven with air flow at 35°C. The dried rhizomes were later ground to a coarse powder, given the voucher specimen number of WK_ZO_052302, and used for the extraction in the Accelerated Solvent Extractor (ASE) (Dionex, Sunnyvale, CA, USA). The 100 mL stainless steel vessel was filled with 20 g of rhizome powder and extracted in 4 cycles of 10 min each using 96% ethanol, at a temperature of 80°C, with flush volume settings of 60% and purge time of 40 s. The obtained extract was later evaporated to dryness at 45°C using a rotary evaporator with a chiller and vacuum pump. The dried residue was subjected to the fractionation using a centrifugal partition chromatograph (CPC) by Armen (Saint Ave, France) equipped with a 250 mL stainless steel column, a UV detector, a quaternary solvent pump and a fraction collector. The fractionation of extract was performed based on the injection of 1 g of dried extract each time, which was dissolved in a 50:50 (v/v) mixture of the upper and lower phases, and was conducted in the descending mode using the gradient separation protocol. First the biphasic solution was prepared from the mixture of hexane:ethyl acetate:methanol:water of 8:2:4:6 (v/v/v/v) and the stationary upper phase was pumped into the column. The sample (dissolved in 6 mL of the aforementioned solution) was injected on a rotating column (1300 rpm) together with the mobile lower phase and was pumped at 4 mL/min for 60 min. Later, the mobile phase was substituted with the upper phase obtained from the 8:2:5:5 (v/v/v/v) mixture of the same solvents and the analysis was performed over the following 30 min. In the end, the third solvents mixture was introduced, at the ratio of 8:2:6:4 (v/v/v/v) and the fractionation with this upper phase lasted for the next 30 min. Eight-milliliter fractions were collected throughout the run and analysed by TLC chromatography (NP TLC plates developed with 10:90 v/v methanol: dichloromethane mixture) for the presence of 6-gingerol, in relation to a solution of a reference 6-gingerol (95% purity, purchased at Sigma Aldrich). The fractions containing 6-gingerol were collected together, evaporated to dryness and used for the further fractionation in a preparative chromatograph.

The purification of 6-gingerol-rich fraction was achieved on a preparative HPLC chromatograph by Shimadzu (Kyoto, Japan) equipped with a quaternary solvents pump (LC-20AP), a column oven (CTO-40C), a PDA detector (SPD-M40), and a fraction collector/autosampler (LH-40) and using a chromatographic column from dr. Maisch: ReproSil-Pur 120 C18-AQ (250 mm x 20 mm, 5.0 µm) (Tubingen, Germany). The applied isocratic mobile phase (12 mL/min) for the analysis was acetonitrile/water (65:35, v/v), and the detection was performed at the wavelength of 282 nm.

The final purity of the isolate was confirmed using the HPLC coupled with an ESI-QTOF-MS mass detector by Agilent Technologies (Santa Clara, CA, USA) that was composed of an HPLC chromatograph (1200 Series) with a binary pump, a degasser, an autosampler, a column thermostat, a UV detector and a QTOF-MS mass spectrometer (G6530B). During the analysis Zorbax Eclipse Plus chromatographic column by Agilent Technologies was used (150 × 2.1 mm, 3.5 µm), the flow rate was set at 0.2 mL/min, the gradient of 0.1% formic acid in acetonitrile (solvent B) in 0.1% aqueous solution of formic acid was as follows: 0-2 min – 1 % B, 3 min – 30% B, 13 min – 45% B, 18 min – 65% B, 28 min – 80% B, 30-35 min – 95% B, 36-45 min – 1% B. Mass spectrometer settings included: 275 and 325°C of gas and sheath gas temperatures, 12 L/min of gas flows, 3000 V of capillary voltage, 10 and 20 V of collision energies CID, 110 V of fragmentor voltage, 1000 V nozzle voltage, 65 V of skimmer voltage, 100-1200 m/z of mass range, and 35 psig of atomizer pressure. Mass Hunter Workstation (B.12.00) by Agilent Technologies was used to process the obtained data.

**Bioanalytical method**

Before the analysis, the samples were prepared for the extraction of 6-gingerol. Frozen brains were transferred one by one to separate Eppendorf vials of 2 mL volume and homogenized with 400 µL 70% acetonitrile of HPLC grade, on ice. The homogenates were centrifuged at 12500 rpm at 5°C (Eppendorf Centrifuge 5702) for 30 min and supernatants were filtered through nylon syringe filters with pore diameter of 0.1 µm. The filtrate was kept frozen in –80°C overnight and on the next day it was centrifuged again, filtered as described above to the autosampler vials and immediately used for chromatographic analysis.

The analysed serum was mixed with cold methanol in equal volumes (200 µL of serum with 200 µL of methanol) and frozen overnight. On the next day it was centrifuged at 12500 rpm at 5°C for 30 min and filtered through nylon syringe filters with pore diameter of 0.1 µm. The samples were used for the chromatographic analysis immediately after preparation.

A chromatographic platform consisting of an ESI-QTOF-MS/MS mass detector coupled with an HPLC chromatograph (1200 series) produced by Agilent Technologies was used to analyze the 6-gingerol content in the biological samples, following the method described above in the section: ‘Isolation of 6-gingerol’. The same method was applied with only a different chromatographic column used, but with the same parameters as the aforementioned one, namely ReproSil XR 120 C18 (150 x 2.1 mm, 3.5 µm) by dr. Maisch (Ammerbuch, Germany).

The concentrations of 6-gingerol in brain and serum samples were calculated based on the calibration curve equation obtained from a standard of 6-gingerol (>95% purity, Sigma Aldrich, St. Louis, CA, USA) injected under the same conditions in a concentration range of 0.001 – 0.1 mg/ml. All injections were made in triplicate.
